# Effects of Thermal Sterilization Conditions on Flavor and Lipid Oxidation of Sauced Duck Necks

**DOI:** 10.3390/foods14173136

**Published:** 2025-09-08

**Authors:** Beibei Chu, Chao Zhang, Yushen Song, Hui Zhou, Xingguang Chen, Qianhui Gu

**Affiliations:** 1 Engineering Research Center of Bio-Process, Ministry of Education, Hefei 230601, China; 19850168519@163.com (B.C.); 19903731586@163.com (C.Z.); xamdmcjy2025@163.com (Y.S.); zhouhui@hfut.edu.cn (H.Z.); 2 School of Food and Biological Engineering, Hefei University of Technology, Hefei 230601, China

**Keywords:** organoleptic properties, peroxide value, thiobarbituric acid-reactive substances, volatile flavor compounds, free fatty acids, correlation analysis

## Abstract

This study aimed to investigate the effect of thermal sterilization on the volatile flavor of sauced duck necks. The study revealed that thermal sterilization significantly reduced the content of unsaturated fatty acids (e.g., oleic acid C18:1n9c) in sauced duck necks. This was accompanied by elevated thiobarbituric acid reactive substances (max 0.86 mg/100 g) and peroxide values (max 1.13 g/100 g), indicating intensified lipid oxidation. Through PLS-DA, six key differential free fatty acids distinguishing the sterilization treatment groups were identified: cis-9-tetradecadecarbonate, methyl tridecarbonate, cis-10-17-cetenoic acid, antioleic acid, cis-13-docosaenoic acid methyl ester, and lauric acid. The primary volatile flavor compounds in sauced duck necks were identified as alkenes and ethers. Post-sterilization alterations in volatile flavor profiles primarily resulted from compositional changes in alkenes, esters, and ethers within the total volatile compounds. Moreover, it was demonstrated that sterilization temperature exerted a significantly greater impact on the quality of sauced duck necks than sterilization duration. Following organoleptic evaluation, samples subjected to low-temperature prolonged sterilization (90 °C for 30 min) exhibited the highest aroma scores, establishing this protocol as the optimal thermal sterilization condition. This study is of great significance for selecting thermal sterilization conditions and maintaining meat flavor.

## 1. Introduction

Sauced duck necks are a representative Chinese dish, favored by consumers for their distinctive flavor, delicious taste, crispy texture, and richness in collagen protein and minerals [1,2]. High-temperature sterilization was often used to achieve commercial sterility after packaging to extend the shelf-life of sauced duck neck products [3]. However, during actual production processes, the flavor of meat products is often compromised by high-temperature sterilization [4]. For instance, the intense heat can cause excessive lipid oxidation, resulting in undesirable off-flavors such as paint-like, cardboard-like, or musty odors [5]. Although the emerging non-thermal sterilization technology in recent years can effectively improve this issue [6,7,8], its relatively high cost and requirements for food shape limit its widespread application [9]. Therefore, the flavor control of thermal sterilization products was still an urgent problem to be solved. However, there are relatively few reports on the impact mechanisms of thermal sterilization on the volatile flavor compounds of sauced duck necks, which makes it difficult to achieve precise control over its flavor profile. This severely limits the rapid development of relevant industries.

Lipid is an important flavor precursor and flavor contributor, which affects the flavor and palatability of meat. It was reported that more than 50% of flavor compounds in cooked meat come from lipid oxidation, indicating that moderate oxidation was helpful for flavor formation of meat products during processing [10]. Previous studies have shown that high-temperature treatment can cause excessive oxidation of lipids [11] and a series of chemical changes of saturated fatty acids (SFA), unsaturated fatty acids (UFA), and free amino acids, which might have a negative impact on the products [12,13,14]. However, in the process of high-temperature sterilization, it is inevitable that the packaged products will have a warmed-over flavor due to lipid oxidation, which seriously affects the flavor of meat products [15]. Zhao et al. [16] investigated the formation pathways of lipid-derived odor-active volatile compounds in Tibetan pork under three cooking methods by examining the pro-oxidative role of free iron and lipid molecular changes. The study revealed that lipid-derived volatiles in low-temperature cooking developed in two distinct stages: initially generated from free fatty acids and lysophosphatidylcholine, followed by phospholipid oxidation in the later stage. In contrast, high-temperature methods triggered rapid lipid oxidation at the onset of cooking, producing characteristic aromas from eicosanoids, triglycerides, and phospholipids. Xia et al. [17] reported the relationship between lipid oxidation and the flavor of different kinds of ducks. Distinct compositional differences in volatile compounds were observed across four sauced duck products, with 19 potential characteristic biomarkers identified. The study found that these 19 characteristic markers exhibit correlations with free fatty acids. For instance, C_18:3_, C_20:2_, and C_20:4_ directly affect the production of 1-octen-3-ol, while C_18:2_, C_20:3_, and C_22:4_ significantly influence the content of nonanal.

Free fatty acids give a rich flavor to duck necks. Therefore, we preliminarily speculate that under different thermal sterilization conditions, the free fatty acids in sauced duck necks will undergo significant changes, which in turn lead to alterations in flavor quality. Consequently, this study aimed to compare the effects of thermal sterilization conditions at different times or temperatures on lipid oxidation and flavor of sauced duck necks. From an academic perspective, this work provides a theoretical basis for the precise regulation of flavor in spiced duck neck. From an industrial production perspective, the optimized thermal sterilization parameters developed in this study can be integrated into production lines to enhance product acceptability and serve as a reference for other meat products requiring high-temperature sterilization.

## 2. Materials and Methods

### 2.1. Sample Preparation

The raw material of duck necks with an age of 45 days was collected from Zhongke Jinrun Food Co., Ltd. (Heze, China). The duck necks (approximately 160–180 g each, yielding a total of 10 kg) from ducks of the same production lot were separated and immediately transported to the laboratory via cold chain.

Before cooking, duck necks were defrosted in flowing clear water for 1~2 h. The duck necks were blanched in hot water (95~100 °C) for 8~12 min. Then, the duck necks were put inside the seasoned brine and boiled at 90~100 °C for 30 min. The seasoned brine (soup) was prepared by mixing 1000 g of water, 100 g sugar, 20 g salt, 50 g monosodium glutamate, 20 g chicken essence, 15 g pepper, 5 g prickly ash, 20 g edible oil, 20 g soy sauce, 2 g star anise, 3 g amomum villosum, and 2 g of angelica dahurica. The duck necks were vacuum-packed after marinating, and samples were divided into four groups for thermal sterilization: unsterilization (YB1), 90 °C for 15 min (YB2), 90 °C for 30 min (YB3), 121 °C for 15 min (YB4). The samples were stored at 4 °C for further analyses. All sterilization conditions have been proven effective by the production facility in eliminating microorganisms in sauced duck necks.

### 2.2. Lipid Oxidation

#### 2.2.1. Peroxide Value

The peroxide value of the duck necks was determined according to the titration method in GB 5009.227-2023 [18]. In brief, a 5.0 g meat sample was mixed with 15.0 g of petroleum ether and allowed to stand for 12 h before being filtered through a funnel with anhydrous sodium sulfate and evaporated in a water bath at 40 °C by rotation. The 2.0 g sample was mixed with 30.0 mL of trichloroacetic acid and glacial acetic acid solution (2:3, *v*/*v*), as well as 1 mL of saturated potassium iodide, and agitated for 30 s before reacting in the dark for 3 min. Finally, add 100 mL of distilled water and 1 mL of starch indicator, and titrate with sodium thiosulfate (0.002 M) until the color turns light yellow and fades stably for 30 s. The following formula is used to calculate peroxide values:

$$POV\left(g/100\;g\right)=\frac{\left(v_{1}-v_{2}\right)\times c\times 0.1269}{m}\times 100$$

Here, *c* (mol/L) refers to the concentration of sodium thiosulfate in the standard titration solution, *m* (g) refers to the mass of the sample, and *v* (mL) refers to the titration volume.

#### 2.2.2. Thiobarbituric Acid-Reactive Substances

The determination of thiobarbituric acid reactants was in accordance with the method described by Angeletti et al. [19] with slight modification. In brief, the duck neck’s meat (5.0 g) was shocked with 50 mL of 7.50% trichloroacetic acid (containing 0.1% EDTA) for 30 min. Then, 5 mL of filtered supernatant was mixed with 5.0 mL of 0.02 M 2-thiobarbituric acid solution and heated in a 90 °C water bath for 40 min, followed by cooling with water. After centrifugation (223.60× *g*, 10 min), 5.0 mL of supernatant was added and mixed with 5.0 mL of chloroform. The absorbance of the supernatant was measured at 532 nm and 600 nm, respectively. Thiobarbituric acid reactive substances (TBARS) values were expressed as malondialdehyde content (mg/100 g sample). The calculation was conducted using the following formula:



T
B
A
R
S (mg100 g)=
A
532−A600155×72.6×110×100

In the formula, A represents absorbance.

### 2.3. Free Fatty Acid Composition

The method of fat extraction was modified according to Ying et al. [20]. A total of 5.0 g of minced duck necks were homogenized in 30 mL of mixed chloroform and methanol (Aladdin reagent (Shanghai) Co., Ltd. (Shanghai, China)) solution (2:1, *v*/*v*) and kept for 30 min before filtering. In the process of sample homogenization, 1 mL of heptadecanoic acid (0.5 mg/mL in methanol containing 0.1% formic acid) was added as an internal standard. The filtrate was then mixed with 10.0 mL of saline (7.3 g/L NaCl and 0.5 g/L CaCl_2_) and centrifuged (2739.10× *g*, 5 min) to absorb the upper liquid. Under a water bath at 44 °C, the sample was vacuum concentrated to obtain the lipid. An amount of 1 mL of dichloromethane was used to activate the aminopropyl silica microcolumn (Agela Technologies Inc., Tianjin, China). Then, 20.0 mg of total lipid was dissolved in 1 mL of chloroform, passed to an aminopropyl silica microcolumn, and eluted to obtain the free fatty acid using 2.0 mL of chloroform and isopropanol (Aladdin reagent (Shanghai) Co., Ltd.) (2:1, *v*/*v*) and 3 mL of 2% acetic acid ether (m/m). The free fatty acid was dried with nitrogen before being mixed with 2.0 mL of 14 % boron trifluoride methanol, 1.0 mL of water, and 1.0 mL of *n*-hexane (mass ratio).

An amount of 1 µL of sample was analyzed by gas chromatography with a SP-2560 capillary column (100 m × 0.25 mm × 0.20 µm). Nitrogen was used as carrier gas with a flow rate of 1 mL/min. The temperature of the injection port was 200 °C, and the split ratio was 25:1. The temperature of the FID detector was 300 °C. The oven conditions were set as follows: the initial temperature was 100 °C for 1 min, ramped to 180 °C at 10 °C/min for 5 min, ramped to 200 °C at 1 °C/min, and kept for 5 min, then it was raised to 230 °C at 10 °C/min and kept for 16 min.

### 2.4. Volatile Flavor Compounds

HS-SPME extraction was performed by the method of Zhou et al. [21] with some modifications. A total of 3.0 g of boneless minced meat and 10 µL of o-dichlorobenzene (64.80 mg/L in methanol) internal standard solution were put into a 20 mL sample bottle. The sample was equilibrated at 50 °C for 1 h in a water bath. The volatiles were extracted by 70 µm PDMS/DVB at 50 °C for 40 min. Then the extraction head was inserted into the GC-MS injector for desorption for 2 min, and the injection temperature was 250 °C.

Chromatographic conditions: Volatiles were separated through TG-5MS capillary column (30 m × 0.25 mm × 0.25 µm). The sample was injected in splitless mode. Helium was used as a carrier gas with a flow rate of 1.2 mL/min. The oven temperature conditions were first isothermal for 2 min and then raised to 100 °C for 1 min at 3 °C/min, raised to 160 °C for 1 min at 5 °C/min, and raised to 280 °C for 1 min at 10 °C/min.

The mass spectrometry (Thermo Scientific, TSQ 8000EVO, Waltham, MA, USA) conditions: The ion source was an electron impact ion source (EI), and the transmission line temperature was 280 °C. The ion source temperature and electron energy were 300 °C and 70 eV, respectively. Full scan acquisition mode was used at a range of 33–450 amµ. Volatile compounds were identified by comparing their mass spectra (MS) and retention indices (RI) with those found in the Wiley 7.0/NIST 105 libraries. Further, some compounds were positively identified by their corresponding chemical standards when they were available. Linear retention indices calculated for standard C8-C20 alkanes (Fluka, Brienz, Switzerland) and n-hexane and heptane (Sigma-Aldrich, St. Louis, MO, USA) were used for confirmation.

### 2.5. Sensory Evaluation

The method of sensory evaluation was modified by referring to Huang et al. [22]. The participants were food professionals, including five males and five females. Participants were trained three times for 30 min each time, focusing on basic sensory training of duck necks in sauce colour, smell, texture, and taste as the evaluation criteria. Four groups of sauced duck necks with different thermal sterilization conditions were evaluated, and the evaluation indexes were as follows: for colour, 1 = dark or dark brown color, no luster, 10 = brown color of marinated meat, glossy; for smell, 1 = faint odour, uncoordinated smell, 10 = strong sauce bittern flavor, overall coordinated smell; for texture, 1 = rough, 10 = smooth; and for taste, 1 = excessive sweetness or spiciness, 10 = sweet moderate spicy. The samples were neatly placed on the white ceramic plate, and all samples were marked with irregular serial numbers to avoid the subjective impression of serial numbers on the evaluators. Between sample evaluations, the evaluators were sent water to rinse their mouths.

### 2.6. Statistical Analyses

Statistical Product and Service Solutions 24.0 was used to analyze the significance of the data. The results were expressed as mean ± standard error (SE) and analyzed by one-way ANOVA (Duncan) for statistical significance. To control the false discovery rate, the Benjamini–Hochberg method was applied to further validate the data (q = 0.05). The chart was analyzed by Origin 8.5 software. Variable importance in projection (VIP) analysis was employed to assess the importance of major flavor precursors, and those compounds with VIP > 1.0 were considered to be statistically significant [23]. Principal component analysis (PCA) was performed by Unscrambler X 4.0. Partial least squares discriminant analysis (PLS-DA) and the VIP analyses were performed by MetaboAnalyst 4.0 [24,25]. All experiments were performed with three biological replicates.

## 3. Results and Discussion

### 3.1. Changes in the Sensory Evaluation Under Different Thermal Sterilization Conditions

**Figure 1:**
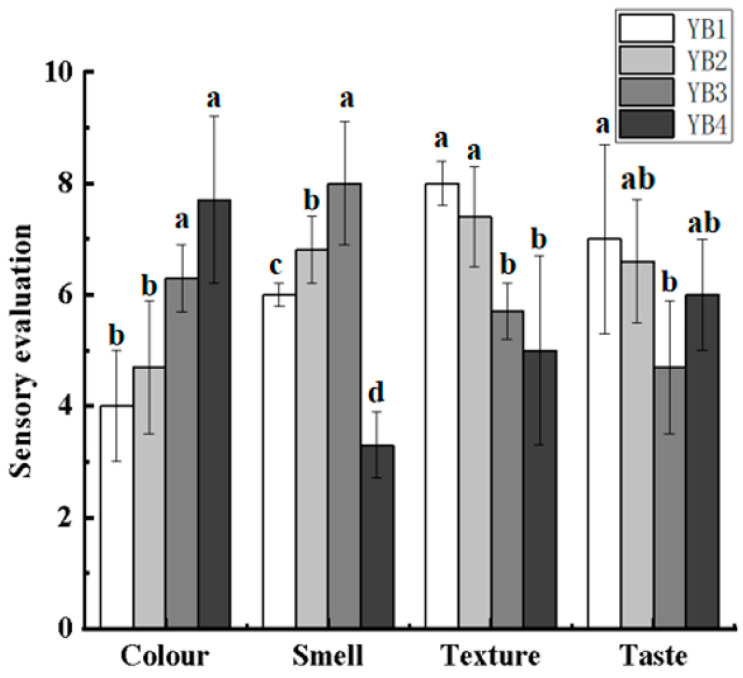
Effects of different thermal sterilization conditions on the sensory evaluation of sauced duck necks. YB1, YB2, YB3, and YB4 mean Unsterilized, Sterilized at 90 °C for 15 min, Sterilized at 90 °C for 30 min, and Sterilized at 121 °C for 15 min, respectively. Significance letters are used solely for within-group comparisons.

The sensory evaluation mainly evaluated the colour, smell, texture, and taste of the samples (Figure 1). After sterilization, the scores of colour of sauced duck necks were significantly higher, and the scores of smell and texture scores were obviously lower, which may be due to the protein precipitation in duck necks caused by high temperature and the fusion with spices and other substances on the surface to form a better sauce color. When the sterilization time was extended from 15 to 30 min, the scores of colour and smell increased, while the texture score decreased significantly (*p* < 0.05) from 0.74 to 0.57. When the sterilization temperature increased from 90 to 121 °C, the scores of smell and texture decreased significantly, and the colour score increased significantly (*p* < 0.05). This occurs as low-temperature sterilization facilitates the controlled degradation of proteins and lipids, generating favorable flavor compounds. In contrast, high temperatures induce excessive oxidation of lipids and proteins, resulting in flavor deterioration [26]. The YB1 group has a worse smell, which may be due to the fact that the meat cannot produce a better smell without heat treatment. In terms of texture, the YB1 group was better than the other three groups for its good chewiness and elasticity. In contrast, the thermal sterilization groups had a soft texture and poor elasticity, which was due to the damage in the structure of muscle protein by thermal treatment [27]. After strong thermal treatment, the microstructure of the YB4 group was dispersed, which was conducive to the infiltration of taste substances in brine. For the set of bactericidal time variables, the scores of colour and smell increased significantly, while the texture score decreased significantly. This means that the chemicals in the duck necks need enough time to react. It was worth noting that there was no significant difference in taste scores of sauced duck necks under different thermal sterilization conditions, which is because the taste is largely determined by the brine rather than the duck itself.

### 3.2. Changes in Lipid Oxidation Under Different Thermal Sterilization Conditions

**Figure 2:**
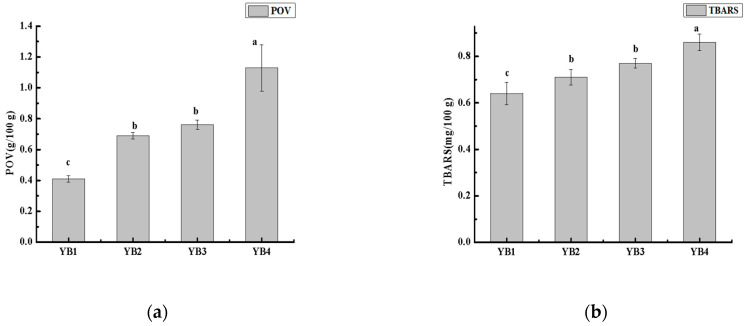
Effects of different thermal sterilization conditions on primary and secondary oxidation of lipid in sauced duck necks. (**a**) POV value of duck necks from different thermal sterilization conditions. POV, Peroxide value. YB1, YB2, YB3, YB4 means Unsterilized, Sterilized at 90 °C for 15 min, Sterilized at 90 °C for 30 min, and Sterilized at 121 °C for 15 min, respectively. (**b**) TBARS value of duck necks from different thermal sterilization conditions. TBARS, thiobarbituric acid reactive substances. YB1, YB2, YB3, and YB4 mean Unsterilized, Sterilized at 90 °C for 15 min, Sterilized at 90 °C for 30 min, and Sterilized at 121 °C for 15 min, respectively. Significance letters are used solely for within-group comparisons.

Thermal sterilization leads to lipid oxidation reaction. Primary lipid peroxidation products include hydroperoxides that are unstable and decompose to generate various secondary products, such as aldehydes [28,29]. This may lead to the flavor change of sauced duck necks. As shown in Figure 2, as expected, thermal sterilization significantly (*p* ˂ 0.05) increased both POV and TBARS. Compared with the unsterilized group (YB1), the POV and TBARS values of sterilization groups (YB2, YB3, and YB4) were significantly higher, which was consistent with the current report [4]. For the effect on the sterilization time, when the sterilization temperature was fixed at 90 °C, with the extension of the sterilization time, the POV range was 0.69~0.76 g/100 g, the TBARS range was 0.71~0.77 mg/100 g, and the difference was not statistically significant (*p* ˂ 0.05). Such findings suggested that lipid oxidation also occurred at low temperatures [30]. However, for the effect on the sterilization temperature, when the sterilization time was fixed at 15 min, with the increase in the sterilization temperature, POV significantly increased from 0.69 (YB2) to 1.13 g/100 g (YB4), and TBARS content significantly increased from 0.71 (YB2) to 0.86 mg/100 g (YB4). When the lipid in the duck necks was oxidized, unsaturated fatty acids form hydrogen peroxide, which then decomposes into secondary products, including malondialdehyde (MDA) and other carbonyl compounds, which could lead to bad flavor [31]. In summary, heat sterilization can lead to oxidation of duck neck lipid, especially under high temperature conditions, which affects the flavor of duck neck.

### 3.3. Changes in Free Fatty Acid Under Different Thermal Sterilization Conditions

Oxidation of free fatty acids could be responsible for volatile compounds in meat products, such as aldehydes, ketones, and alcohols, which were believed to be the main cause of sensory degradation of cooked meat [32]. The free fatty acid composition of sauced duck necks under different thermal sterilization conditions is shown in Table 1. In total, 18 kinds of fatty acids were detected in four samples, including 11 kinds of saturated fatty acids and 7 kinds of unsaturated fatty acids. Palmitic acid (C_16:0_), stearic acid (C_18:0_), and octadecenoic acid (C_18:1n9c_) were the main free fatty acids of sauced duck necks, which were in agreement with the findings of Wang et al. [33]. Moreover, elaidic acid (C_18:1n9t_), erucic acid (C_22:1n9_), and cis-5,8,11,14,17-eicosapentaenoic acid (C_20:5n3_) were also found in low concentrations.

Compared with the unsterilized group (YB1: 260.56 mg/100 g), the saturated fatty acid content of the sterilization groups (YB2: 398.32 mg/100 g, YB3: 308.32 mg/100 g, and YB4: 345.24 mg/100 g) was significantly higher. However, the content of unsaturated fatty acids in YB2 (158.55 mg/100 g), YB3 (129.42 mg/100 g), and YB4 (102.36 mg/100 g) was significantly lower than that of YB1 (270.44 mg/100 g). It was easier to be oxidized if the ratio of polyunsaturated fatty acids (PUFA) to saturated fatty acids in meat was higher [34]. Among these unsaturated fatty acids in sterilization groups, oleic acid (C_18:1n9c_) accounted for the largest proportion of total free fatty acids, which were 9.70%, 12.70%, and 12.30%, respectively. The content of unsaturated fatty acids decreased significantly (*p* < 0.05) after thermal sterilization, among which cis-9-tetradecadecarbonate (C_14:In5_) and octadecenoic acid (C_18:In9c_) decreased significantly. Because unsaturated fatty acids react with molecular oxygen via a free radical mechanism, the hydroperoxides formed were regarded as principal oxidation products [35]. When the sterilization temperature was fixed at 90 °C, with the extension of the sterilization time, the saturated fatty acids and unsaturated fatty acids content of sauced duck necks decreased significantly. After 30 min, only 12 free fatty acids were detected, among which butyric (C_4:0_), capric (C_10:0_), undecanoic (C_11:0_), tridecanoic (C_13:0_), myristic (C_14:0_), and cis-9-tetradecadecarbonate (C_14:1n5_) were not detected. After sterilization at 121 °C for 15 min, the content of unsaturated fatty acids in the neck of sauced ducks decreased further, and only nine kinds of free fatty acids were found, mainly includes pentadecanoic (C_15:0_), palmitic (C_16:0_), palmitoleic acids (C_16:1n7_), cis-10-17-cetenoic acid (C_17:1n7_), stearic (C_18:0_), octadecenoic acid (C_18:1n9c_), arachidic (C_20:0_), tricosanoic (C_23:0_) and cis-5,8,11,14,17-eicosapentaenoic acid (C_20:5n3_).

Interestingly, as important flavor precursors, there was no doubt that the content of unsaturated fatty acids decreases with the increase in thermal sterilization temperature or time, while the content of saturated fatty acids increases during this processing. For example, the content of saturated fatty acids in the YB4 group was higher than that in the YB1 group, among which pentadecanoic (C_15:0_), palmitic (C_16:0_), stearic (C_18:0_), arachidic (C_20:0_), and tricosanoic (C_23:0_) increased significantly. Similar changes were observed in the YB2 and YB3 groups, among which lauric (C_12:0_), pentadecanoic (C_15:0_), palmitic (C_16:0_), stearic (C_18:0_), arachidic (C_20:0_), and tricosanoic (C_23:0_) increased significantly. This may be caused by the oxidation of unsaturated fatty acids [36]. Saturated fatty acids were generally considered to have a negative impact on food volatile components [37]. It is worth noting that the saturated fatty acid content of the YB3 group (308.32 mg/100 g) was the lowest among all the sterilization groups, which may be one of the reasons for its best smell score in the sensory evaluation experiment.

In summary, different heat treatment conditions altered the composition of free fatty acids. Heat treatment promoted the degradation of unsaturated fatty acids such as cis-9-tetradecenoate (C_14:1n5_), oleic acid (C_18:1n9c_), and elaidic acid (C_18:1n9t_), while increasing the content of saturated fatty acids. This had a negative impact on the flavor of sauced duck necks. Low-temperature heat treatment resulted in less fatty acid degradation, which is beneficial for the formation of duck neck aroma. Both heat treatment temperature and time significantly affected the free fatty acid composition of the duck neck. Combining the sensory evaluation results, low-temperature, long-duration heat treatment was identified as the optimal condition.

**Figure 3:**
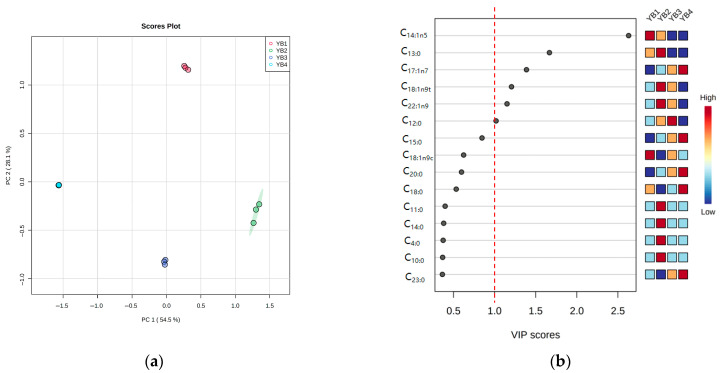
Discriminant analysis of free fatty acids under different thermal sterilization conditions by the partial least squares method. (**a**) In the score plot of PLS-DA, Component 1 accounted for 54.50% of the total variance, and Component 2 accounted for 28.10% of the total variance; (**b**) The important free fatty acids were identified by PLS-DA, and the relative contents of free fatty acids under different thermal sterilization conditions were shown in the color box on the right. The red dotted line indicates that VIP = 1.

PLS-DA was used to treat different thermal sterilization conditions, and fatty acids with significant differences were selected as markers (Figure 3a). Permutation testing with 200-time cross-validation was performed to validate the model’s reliability. The results showed that the R^2^ and Q^2^ intercept values were 0.9122 and −1.8531, respectively, demonstrating a good model fit with no overfitting occurring. Component 1 represents 54.5%, and Component 2 represents 28.1% of the total difference, respectively. The four samples have no overlap and are located in four quadrants. The important results of PLS-DA identification of free fatty acids are shown in Figure 3b. From this, it can be seen that cis-9-tetradecadecarbonate (C_14:1n5_), methyl tridecarbonate (C_13:0_), cis-10-17-cetenoic acid (C_17:1n7_), antioleic acid (C_18:1n9t_), cis-13-docosaenoic acid methyl ester (C_22:1n9_), and lauric acid (C_12:0_) were identified as key free fatty acids, which were the substances with high ability to distinguish among each group. The most obvious change was cis-9-tetradecane-enoic acid methyl ester (C_14:1n5_) in the YB1 group, whose content decreased significantly (*p* < 0.05) after moderate thermal sterilization in the YB2 group, but it was not detected in YB3 and YB4. These findings indicated that thermal sterilization temperature and time have a great influence on the fatty acids. Therefore, considering the effect of free fatty acids on the flavor, the six key fatty acids identified were the main substances for the effect and regulation of thermal sterilization on the flavor of sauced duck necks.

### 3.4. Changes in Volatile Flavor Components Under Different Thermal Sterilization Conditions

The aroma and flavor characteristics of cooked meat were an important basis for consumers to choose meat products. These volatile compounds were produced by thermal degradation of lipids, Maillard reaction, the interaction between Maillard reaction products and lipid oxidation products, and thiamine degradation [4,38].

**Figure 4:**
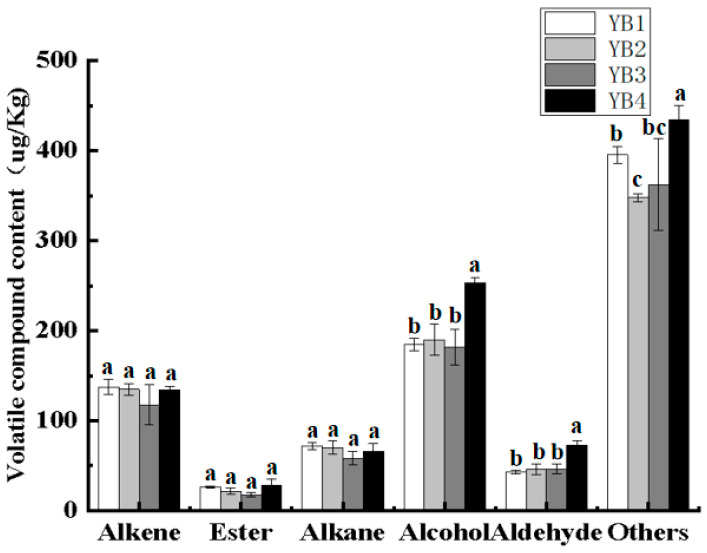
Species and total volatile substances in duck necks under different thermal sterilization conditions. YB1, YB2, YB3, and YB4 mean Unsterilized, Sterilized at 90 °C for 15 min, Sterilized at 90 °C for 30 min, and Sterilized at 121 °C for 15 min, respectively. Significance letters are used solely for within-group comparisons.

HS-SPME-GC-MS was used for qualitative and quantitative analysis of volatile flavor components in sauced duck necks. Figure 4 shows the species and content comparison of volatile substances in sauced duck necks with different thermal sterilization methods. All identified volatiles were categorized into six chemical groups, including alkene, ester, alkane, alcohol, aldehyde, and others. The contents of alkenes and alkanes in the unsterilized group were not significantly different from those in the sterilized group. In contrast, the contents of alcohol and aldehyde significantly increased after high-temperature sterilization. The other substances decreased significantly after low-temperature sterilization and increased significantly after high-temperature sterilization compared with the control group. When the sterilization temperature was fixed at 90 °C, the effect of sterilization time on the contents of volatile substances was compared. The results showed that there was no significant difference in the contents of various types of substances except for other substances. When the sterilization time was fixed at 15 min, the effects of sterilization temperature on the contents of volatile substances in the duck necks were compared. The results showed that the contents of aldehydes, alcohols, and other substances increased significantly, but there was no significant difference between alkenes and esters.

A total of 56 volatile compounds isolated from sauced duck necks are shown in Table 2. The mixture comprised 18 alkenes, 7 aldehydes, 3 alkanes, 12 esters, 11 alcohols, and 5 heterocyclics.

Alkenes were the main flavor compounds in sauced duck necks, accounting for 137.26, 134.92, 132.89, and 134.55 µg/kg of the YB1, YB2, YB3, and YB4, respectively. The results were relatively consistent with those found by Zhou et al. [21], who reported that alkenes occupy the majority of volatile compounds of salami sausages and sauced duck necks, respectively. The content of these substances decreased significantly after high-temperature sterilization, indicating that high-temperature sterilization has a subtractive effect on these substances, and low-temperature sterilization promotes the release of flavor. There were two main sources of alkenes: one was the long-term accumulation in animal feed; the second was from spices added in the processing [39]. Therefore, according to the existing reports, it can be speculated that the main alkenes in braised duck necks included *β*-pinene, caryophyllene, 3-carene, *β*-rolene, and *β*-curcumene, which came from the spices in braised duck soup and had antioxidant effects [40]. It was not surprising that the content of these substances was high because of the added spices in the duck necks. Although the threshold value of alkenes was low, the high relative content of alkenes contributed to the overall flavor attributes of sauced duck necks.

A total of seven aldehydes were detected in the four groups of samples, mainly including hexanal, 4-1-methylethylbenzaldehyde, and (*E,E*)-2,4-decenal, among which (*E,E*)-2,4-decenal, with a lower threshold and higher content, was detected in the YB4 (8.64 µg/kg, OAV: 123.42). In general, all aldehydes have a negative effect on flavor, especially unsaturated aldehydes such as (*E,E*)-2,4-decadienal and (*E*)-2-nonenal, because they have a negative effect on the senses [41]. Hexanal is a metabolite of C_18:1_ and C_18:2_, which is ubiquitous in cooked meat products. It has a grassy taste at low levels and an unpleasant warmed-over flavor at high levels [1]. Therefore, the content of the unsterilized and low-temperature sterilization group is lower than that of the high-temperature sterilization group. Aldehydes were mostly aliphatic; benzaldehyde was the only identified aromatic compound, which was derived from the Strecker degradation of amino acids and has aromatic flavor [42]. Low threshold aldehydes were closely associated with the production of superheated taste after high-temperature sterilization, and their formation in meat products was attributed to the self-oxidation of meat lipids to produce hydroperoxides, which were decomposed into a large number of volatile compounds through many different pathways. Cooked meat was susceptible to lipid oxidation, and phospholipids are major contributors to lipid oxidation and superheated flavor formation [16].

A total of 11 alcohols were detected in four groups. The most abundant alcohols were 3,7-dimethyl-1,6-octandiene-3-alcohols, with the contents of 162.42, 167.56, 161.28, and 188.62 µg/kg in each group, respectively. Its flavor threshold was high; hence, it did not contribute much to the overall flavor. The contents of alcohol in the high-temperature sterilization group were significantly higher than those in the unsterilization and low-temperature sterilization groups. Alcohols were derived from the transformation of fatty acids and are closely related to the formation of cooked meat flavor [43].

A total of 12 esters were detected in four groups of samples, including ethyl decanoate and ethyl hexadecanoate. Esters were aromatic substances generated by the esterification of acids and alcohols. There was no significant difference in ester content between the low-temperature sterilization and unsterilization groups. In contrast, the content of esters decreases significantly after high-temperature sterilization, which was due to the promotion of the Maillard reaction and the degradation of amino acids by appropriate heat treatment [44]. After high-temperature sterilization, the decrease in the smell score of duck necks may be related to the significant decrease in esters.

Other substances detected were the substances with the highest content of total volatile compounds, including anisole and estragole, of which the anisole content was 285.77, 244.21, 267.31, and 336.62 µg/kg in YB1, YB2, YB3, and YB4, respectively. The contents of ether compounds in the sterilization groups were significantly higher than those in the unsterilization group. It has been reported that the anisole was derived from star anise and five-spice powder in brine soup [40].

**Figure 5:**
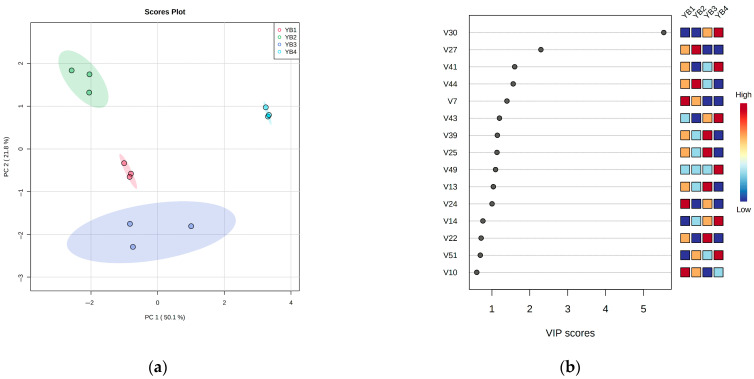
Discriminant analysis of volatile flavor compounds under different thermal sterilization conditions by the partial least squares method. (**a**) In the score plot of PLS-DA, Component 1 accounted for 50.10% of the total variance, and Component 2 accounted for 21.80% of the total variance; (**b**) The important volatile flavor compounds were identified by PLS-DA, and the relative contents of flavor compounds under different thermal sterilization conditions were shown in the color box on the right.

From the PLS-DA of Figure 5a, it can be seen that there was no overlap among the four groups of samples. Permutation testing with 200-time cross-validation was performed to validate the model’s reliability. The results showed that the R^2^ and Q^2^ intercept values were 0.9264 and −2.0126, respectively, demonstrating a good model fit with no overfitting occurring. Component 1 accounts for 50.1%, and Component 2 accounts for 21.8% of the total variance. Therefore, different thermal sterilization conditions have a significant impact on the flavor. The results of PLS-DA identification of important volatile compound were shown in Figure 5b, the higher the VIP score was, the stronger the ability to distinguish the key volatile compounds identified, including 11 substances such as V30 (ethyl oleate), V27 (tetradecanoic acid, ethyl ester), V41 (tert-hexadecanethiol), V44 (2-methyl-1-hexadecanol), V7 (cyclohexene, 1-methyl-4-(1-methylethylidene)-), V43 (epiglobulol), V39 (behenic alcohol), V25 (methyl tetradecanoate), V49 ((*E,E*)-2,4-decadienal), V13 (*β*-copaene) and V24 (dodecanoic acid, ethyl ester). From the color gradient on the right side, it can be seen that the relative content of volatile compounds changed significantly with the increase in temperature or time. While the primary flavor compounds may not be the most impactful, significant changes in a few minor substances can also substantially affect flavor [14]. Sensory evaluation confirmed that low-temperature, long-time thermal sterilization (90 °C, 30 min) yielded the highest aroma score, establishing it as the optimal processing condition.

### 3.5. Correlation Between Free Fatty Acids and Volatile Flavor Substances

**Figure 6:**
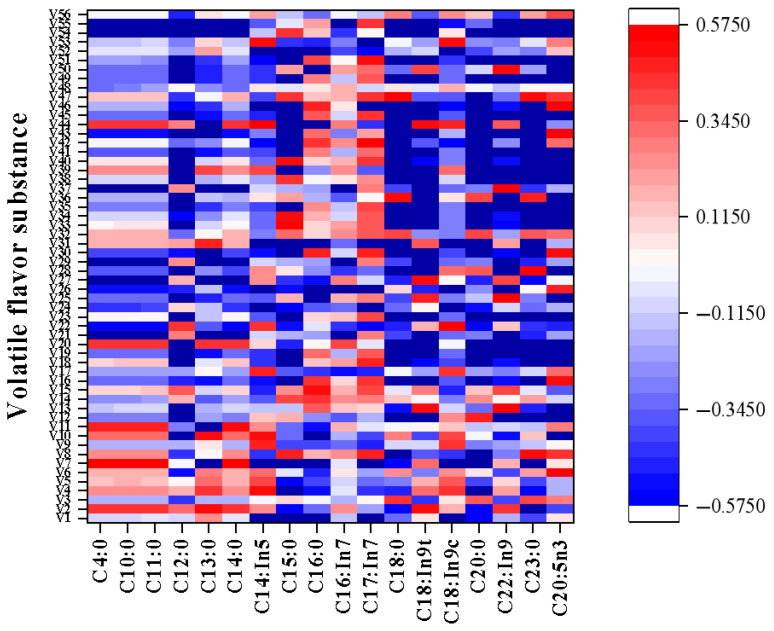
Pearson correlation analysis of free fatty acids and volatile flavor substances in sauced duck necks under different thermal sterilization conditions.

Pearson analysis of the relationship between free fatty acids and volatile flavor substances is shown in Figure 6. Of all the compounds produced during oxidation, aldehydes are considered to be the most important decomposition products and the largest source of volatile flavor in meat [45]. There was a significant negative correlation between hexaldehyde and C_18:1n9t_ (*r* = −0.76, *p* < 0.01). The contents of 4-(1-methylethyl)-benzaldehyde had negative correlations with C_20:0_ (*r* = −0.60, *p* < 0.05). *β*-pinene, which has the most abundant content, was positively correlated with C_23:0_ (*r* = −0.62, *p* < 0.05). There was a significant negative correlation between ethyl hexadecate and C_12:0_ (*r* = −0.79, *p* < 0.01), C_18:1n9t_ (*r* = −0.75, *p* < 0.01), and C_22:1n9_ (*r* = −0.78, *p* < 0.01). There was a significant positive correlation between 3, 7-dimethyl-1, 6-octadiene-3-ol and C_18:0_ (*r* = 0.71, *p* < 0.05), C_20:0_ (*r* = 0.69, *p* < 0.05), C_23:0_ (*r* = 0.66, *p* < 0.05), C_20:5n3_ (*r* = 0.58, *p* < 0.05), and a significant negative correlation between 3,7-dimethyl-1, 6-octadiene-3-ol and C_18:1n9t_ (*r* = −0.58, *p* < 0.05). Estragole and anethole were the most abundant in sauced duck necks. Estragole was negatively correlated with C_17:1n7_ (*r* = −0.58, *p* < 0.05). Anethole was negatively correlated with C_12:0_ (*r* = −0.73, *p* < 0.01), C_13:0_ (*r* = −0.61, *p* < 0.05), C_18:1n9t_ (*r* = −0.88, *p* < 0.01), C_22:1n9_ (*r* = −0.81, *p* < 0.01), and positively correlated with C_18:0_ (*r* = 0.84, *p* < 0.01), C_23:0_ (*r* = 0.82, *p* < 0.01).

## 4. Conclusions

The thermal sterilization resulted in lower content of unsaturated fatty acids, and a higher increase in values of TBARS and POV compared with the unsterilized sauced duck necks group. The oleic acid (C_18:1n9c_) decreased significantly with the increase in temperature or time of thermal sterilization, and the content of saturated fatty acids, such as stearic acid (C_18:0_) and palmitic acid (C_16:0_), increased significantly with the increase in oxidation degree. The main volatile flavor of sauced duck necks was composed of alkenes and ethers. The change of duck necks’ volatile flavors after thermal sterilization was mainly related to the proportion of alkenes, esters, and ethers in total volatile flavor compounds. Six free fatty acids were screened out by the partial least squares (PLS-DA) method and identified as the markers causing the difference between samples. The effect of thermal sterilization temperature on the quality of duck necks was greater than thermal sterilization time. The low temperature and long-time thermal sterilization (90 °C for 30 min) had the highest aroma score, which was the best thermal sterilization condition in this study. These findings, on one hand, offer scientific guidance for precisely controlling the flavor of sauced duck necks through the modification of thermal sterilization conditions or the addition of flavor precursor substances. On the other hand, they provide a theoretical foundation for flavor prediction of sauced duck necks using methods such as machine learning, which may be further applied to actual industrial production in the future.

This study still has certain limitations. The specific mechanism underlying the degradation of free fatty acids and the loss of flavor in sauced duck necks during the sterilization process has not been fully elucidated. In future research, we plan to further investigate this mechanism through aspects such as lipase activity, lipid oxidation pathways, Maillard reaction kinetics, and metabonomics.

## Figures and Tables

**Table 1:** Effects of different thermal sterilization methods on free fatty acids.

		YB1 (mg/100 g)	YB2 (mg/100 g)	YB3 (mg/100 g)	YB4 (mg/100 g)
1	C_4:0_	0 ± 0.00	1.66 ± 0.06	0 ± 0.00	0 ± 0.00
2	C_10:0_	0 ± 0.00	14.86 ± 0.14	0 ± 0.00	0 ± 0.00
3	C_11:0_	0 ± 0.00	7.35 ± 0.87	0 ± 0.00	0 ± 0.00
4	C_12:0_	19.23 ± 0.23 ^c^	41.99 ± 0.26 ^b^	62.54 ± 0.42 ^a^	0 ± 0.00 ^c^
5	C_13:0_	53.75 ± 0.59 ^b^	124.52 ± 0.58 ^a^	0 ± 0.00 ^c^	0 ± 0.00 ^c^
6	C_14:0_	0 ± 0.00	2.11 ± 0.12	0 ± 0.00	0 ± 0.00
7	C_14:1n5_	97.51 ± 1.87 ^a^	23.68 ± 4.07 ^b^	0 ± 0.00 ^c^	0 ± 0.00 ^c^
8	C_15:0_	8.03 ± 0.37 ^d^	14.82 ± 0.62 ^c^	19.36 ± 2.17 ^b^	25.44 ± 1.57 ^a^
9	C_16:0_	73.87 ± 0.61 ^d^	88.37 ± 1.25 ^c^	111.90 ± 0.58 ^b^	101.64 ± 0.68 ^a^
10	C_16:1n7_	15.22 ± 4.46 ^a^	27.34 ± 5.31 ^b^	22.99 ± 3.34 ^b^	19.95 ± 0.25 ^ab^
11	C_17:1n7_	0 ± 0.00 ^b^	16.93 ± 4.02 ^a^	18.07 ± 0.95 ^a^	18.76 ± 0.42 ^a^
12	C_18:0_	47.89 ± 0.65 ^b^	38.88 ± 1.72 ^d^	42.10 ± 0.37 ^c^	101.80 ± 0.46 ^a^
13	C_18:1n9t_	8.81 ± 0.92 ^c^	20.28 ± 1.31 ^a^	17.56 ± 0.58 ^b^	0 ± 0.00 ^d^
14	C_18:1n9c_	138.52 ± 12.10 ^a^	53.99 ± 0.31 ^b^	55.62 ± 0.90 ^b^	54.91 ± 0.16 ^b^
15	C_20:0_	32.86 ± 0.42 ^d^	41.14 ± 0.24 ^c^	46.69 ± 0.63 ^b^	75.60 ± 0.16 ^a^
16	C_22:1n9_	2.20 ± 0.13 ^b^	8.63 ± 0.70 ^a^	8.13 ± 0.47 ^a^	0 ± 0.00 ^c^
17	C_23:0_	24.93 ± 0.53 ^b^	22.62 ± 0.50 ^c^	25.73 ± 1.13 ^b^	40.76 ± 0.23 ^a^
18	C_20:5n3_	8.18 ± 0.28 ^b^	7.70 ± 0.22 ^c^	7.05 ± 0.10 ^d^	8.74 ± 0.11 ^a^
	SFA	260.56 ± 3.40	398.32 ± 6.36	308.32 ± 5.30	345.24 ± 3.10
	UFA	270.44 ± 19.76	158.55 ± 15.94	129.42 ± 6.34	102.36 ± 0.94
	∑FFA	531 ± 23.16	556.87 ± 22.30	437.74 ± 11.64	447.60 ± 4.04
	SFA/UFA	0.96	2.52	2.39	3.38

SFA means Saturated fatty acid; UFA means Unsaturated fatty acid; ∑FFA means Total free fatty acid content. Identification of free fatty acids was carried out by different retention times. For a–d, identical letters in the same row indicate that there was no significant difference in different treatment groups (*p* < 0.05).

**Table 2:** Volatile flavor compounds in duck necks under different thermal sterilization conditions.

Order	Retention Time	Components	Threshold Value (µg/kg)	YB1	OAV	YB2	OAV	YB3	OAV	YB4	OAV
V1	7.04	α-Pinene	ND	7.03 ± 0.93 ^a^		5.36 ± 1.39 ^ab^		5.06 ± 0.89 ^b^		4.73 ± 0.36 ^b^	
V2	8.61	β-Pinene	6	46.81 ± 4.71 ^a^	7.80	49.04 ± 0.78 ^a^	8.17	45.28 ± 7.11 ^a^	7.55	41.26 ± 1.70 ^a^	6.87
V3	9.26	(+)-2-Carene	ND	3.15 ± 0.41 ^a^		3.04 ± 0.07 ^a^		2.99 ± 0.50 ^a^		3.40 ± 0.29 ^a^	
V4	10.03	trans-β-Ocimene	34	10.52 ± 0.15 ^a^	0.31	10.22 ± 0.22 ^a^	0.30	9.60 ± 1.64 ^a^	0.28	9.00 ± 0.62 ^a^	0.26
V5	10.31	3-Carene	5	9.91 ± 0.83 ^a^	1.98	9.62 ± 0.37 ^a^	1.92	9.11 ± 1.51 ^a^	1.82	8.89 ± 0.78 ^a^	1.78
V6	11.05	2-Carene	ND	4.68 ± 0.30 ^a^		4.52 ± 0.22 ^a^		2.65 ± 2.25 ^a^		4.64 ± 0.48 ^a^	
V7	12.07	Cyclohexene, 1-methyl-4-(1-methylethylidene)-	ND	1.94 ± 0.05 ^a^		1.84 ± 0.06 ^b^		ND		ND	
V8	16.97	γ-Elemene	ND	3.51 ± 0.11 ^b^		4.01 ± 0.23 ^ab^		3.56 ± 0.53 ^b^		4.29 ± 0.10 ^a^	
V9	17.84	alfa.-Copaene	ND	3.04 ± 0.74 ^a^		0.42 ± 0.07 ^a^		0.31 ± 0.10 ^a^		0.49 ± 0.02 ^a^	
V10	18.81	Caryophyllene	64	18.82 ± 0.53 ^a^	0.29	17.86 ± 0.97 ^ab^	0.27	16.63 ± 2.43 ^c^	0.26	16.93 ± 0.31 ^b^	0.26
V11	19.08	trans-α-Bergamotene	ND	0.78 ± 0.05 ^ab^		0.85 ± 0.13 ^a^		0.63 ± 0.08 ^b^		0.75 ± 0.10 ^ab^	
V12	19.52	Humulene	ND	1.23 ± 0.18 ^ab^		1.06 ± 0.03 ^b^		0.96 ± 0.10 ^b^		1.39 ± 0.15 ^a^	
V13	19.97	γ-Muurolene	ND	2.71 ± 0.17 ^b^		2.69 ± 0.36 ^b^		8.48 ± 5.76 ^a^		—	
V14	19.71	β-copaene	ND	0.30 ± 0.01 ^a^		0.39 ± 0.09 ^a^		1.28 ± 0.78 ^a^		0.93 ± 0.19 ^a^	
V15	20.21	α-Guaiene	ND	4.32 ± 0.20 ^a^		5.76 ± 1.63 ^a^		6.49 ± 2.34 ^a^		5.43 ± 0.14 ^a^	
V16	20.32	β-curcumene	ND	9.72 ± 0.96 ^b^		10.51 ± 1.59 ^b^		13.29 ± 8.24 ^b^		23.15 ± 2.86 ^a^	
V17	20.58	β-Bisabolene	ND	7.00 ± 3.05 ^a^		5.03 ± 0.52 ^a^		4.97 ± 0.56 ^a^		5.34 ± 1.26 ^a^	
V18	21.35	α-Calacorene	ND	1.79 ± 0.30 ^c^		2.70 ± 0.65 ^b^		1.60 ± 0.14 ^c^		3.93 ± 0.47 ^a^	
V19	11.92	Octanoic acid, methyl ester	ND	1.64 ± 0.06 ^b^		1.64 ± 0.06 ^b^		1.66 ± 0.36 ^b^		3.60 ± 0.27 ^a^	
V20	16.62	Decanoic acid, methyl ester	ND	2.10 ± 0.08 ^b^		2.77 ± 0.09 ^a^		2.85 ± 0.46 ^a^		0.87 ± 0.04 ^c^	
V21	17.5	(R)-lavandulyl acetate	ND	ND		4.43 ± 0.45		ND		ND	
V22	18.16	Decanoic acid, ethyl ester	5	0.87 ± 0.38 ^a^	0.17	ND		0.88 ± 0.14 ^a^		ND	
V23	21.46	Geranyl isovalerate	ND	0.60 ± 0.18 ^b^		0.88 ± 0.27 ^b^		0.45 ± 0.05 ^b^		1.54 ± 0.26 ^a^	
V24	22.2	Dodecanoic acid, ethyl ester	ND	3.75 ± 2.84 ^a^		ND		1.95 ± 0.21 ^ab^		ND	
V25	24.64	Methyl tetradecanoate	ND	0.41 ± 0.06 ^b^		0.28 ± 0.15 ^b^		5.33 ± 0.24 ^a^		ND	
V26	25.65	Ethyl 9-tetradecenoate	ND	1.81 ± 0.68 ^a^		0.0089 ± 0.004 ^b^		0.23 ± 0.027 ^b^		0.63 ± 0.01 ^b^	
V27	25.86	Tetradecanoic acid, ethyl ester	ND	2.52 ± 0.92 ^b^		7.57 ± 1.03 ^a^		ND		ND	
V28	26.94	Hexadecanoic acid, ethyl ester	2000	12.83 ± 3.80 ^ab^	<0.01	4.61 ± 1.18 ^bc^	<0.01	2.80 ± 1.87 ^c^	<0.01	15.72 ± 7.38 ^a^	<0.01
V29	31.86	Ethyl Oleate	ND	ND		0.23 ± 0.04		ND		ND	
V30	28.86	Ethyl 9-hexadecenoate	ND	ND		ND		1.39 ± 0.38 ^b^		5.27 ± 0.23 ^a^	
V31	8.16	Bicyclo [3.1.0]hexane, 4-methylene-1-(1-methylethyl)-	ND	62.87 ± 2.56 ^a^		55.37 ± 1.54 ^ab^		51.04 ± 7.56 ^b^		43.37 ± 0.42 ^c^	
V32	21.56	Tetradecane, 2,6,10-trimethyl-	ND	4.46 ± 1.61 ^b^		7.36 ± 1.75 ^a^		4.51 ± 0.04 ^b^		9.01 ± 1.00 ^a^	
V33	22.27	Nonadecane	ND	4.55 ± 2.19 ^b^		7.43 ± 4.37 ^b^		2.66 ± 0.41 ^b^		13.88 ± 2.07 ^a^	
V34	11.44	1,6-Octadien-3-ol, 3,7-dimethyl-	ND	162.42 ± 6.77 ^b^		167.56 ± 14.28 ^ab^		161.28 ± 18.32 ^b^		188.62 ± 2.80 ^a^	
V35	13.27	Terpinen-4-ol	340	7.32 ± 0.28 ^b^	0.02	7.37 ± 0.87 ^b^	0.02	7.28 ± 0.76 ^b^	0.02	10.06 ± 0.07 ^a^	0.03
V36	13.6	α-Terpineol	ND	1.66 ± 0.45 ^a^		1.54 ± 0.20 ^a^		1.2 ± 0.14 ^a^		1.86 ± 0.08 ^a^	
V37	19.23	α-acorenol	ND	ND		1.64 ± 0.61		ND		ND	
V38	21.67	1,6,10-Dodecatrien-3-ol, 3,7,11-trimethyl-, (E)-	ND	5.34 ± 0.70 ^b^		5.44 ± 0.87 ^b^		4.77 ± 0.32 ^b^		6.75 ± 0.45 ^a^	
V39	21.88	Behenic alcohol	ND	0.54 ± 0.05 ^a^		0.51 ± 0.10 ^a^		0.56 ± 0.16 ^a^		ND	
V40	22.07	(-)-Spathulenol	ND	1.28 ± 0.22 ^b^		1.50 ± 0.31 ^ab^		1.19 ± 0.15 ^b^		1.91 ± 0.21 ^a^	
V41	22.43	tert-Hexadecanethiol	ND	2.92 ± 0.99 ^b^		1.17 ± 0.58 ^b^		1.19 ± 0.08 ^b^		36.12 ± 3.49 ^a^	
V42	22.55	12-Methyl-E,E-2,13-octadecadien-1-ol	ND	1.51 ± 0.52 ^b^		2.54 ± 0.48 ^ab^		2.39 ± 0.80 ^ab^		3.86 ± 1.60 ^a^	
V43	23.26	Epiglobulol	ND	1.01 ± 0.54 ^b^		ND		1.15 ± 0.49 ^b^		3.58 ± 2.01 ^a^	
V44	23.47	1-Hexadecanol, 2-methyl-	ND	0.71 ± 0.48 ^a^		0.71 ± 0.23 ^a^		0.33 ± 0.01 ^ab^		ND	
V45	4.3	Hexanal	4.5	4.50 ± 0.93 ^b^	1	4.58 ± 1.42 ^b^	1.01	5.08 ± 0.67 ^b^	1.13	10.90 ± 1.59 ^a^	2.42
V46	7.9	Benzaldehyde	350	4.16 ± 0.24 ^c^	0.01	5.67 ± 0.33 ^b^	0.02	5.64 ± 0.90 ^b^	0.02	10.11 ± 0.64 ^a^	0.03
V47	14.77	Benzaldehyde, 4-(1-methylethyl)-	24	25.35 ± 0.20 ^a^	1.05	27.41 ± 2.05 ^a^	1.14	25.48 ± 3.09 ^a^	1.06	29.23 ± 1.23 ^a^	1.21
V48	15.58	2-Propenal, 3-phenyl-	15.1	5.86 ± 1.50 ^a^	0.38	4.43 ± 3.93 ^a^	0.29	5.96 ± 0.73 ^a^	0.39	5.37 ± 0.99 ^a^	0.35
V49	16.48	2,4-Decadienal, (E,E)-	0.07	ND		ND		ND		8.64 ± 1.80	123.42
V50	24.64	Methyl tetradecanoate	ND	ND		ND		0.35 ± 0.06		ND	
V51	26.26	Tetradecanal	14	2.74 ± 0.40 ^b^	0.20	3.70 ± 0.61 ^b^	0.26	3.57 ± 0.33 ^b^	0.26	8.45 ± 1.04 ^a^	0.60
V52	11.78	Thujone	360	3.47 ± 0.03 ^a^	<0.01	3.17 ± 0.13 ^a^	<0.01	3.05 ± 0.49 ^a^	<0.01	3.09 ± 0.06 ^a^	<0.01
V53	13.77	Estragole	16	78.33 ± 2.12 ^a^	4.89	65.61 ± 10.16 ^a^	4.10	62.16 ± 16.71 ^a^	3.89	66.93 ± 12.97 ^a^	1.43
V54	15.91	Anethole	15	285.77 ± 5.41 ^b^	19.05	244.21 ± 5.71 ^b^	16.28	267.31 ± 36.41 ^b^	17.82	336.62 ± 20.47 ^a^	22.44
V55	20.06	Benzene, 1-(1,5-dimethyl-4-hexenyl)-4-methyl-	ND	ND		8.14 ± 0.60 ^a^		4.96 ± 2.33 ^b^		ND	
V56	20.39	Naphthalene	6.8	27.78 ± 1.82 ^a^	4.08	26.71 ± 2.66 ^a^	3.92	24.88 ± 2.97 ^a^	3.65	27.94 ± 2.44 ^a^	4.10

Note: ^a–c^ Identical letters in the same row indicate that there was no significant difference in different treatment groups (*p* < 0.05); ND means not detected or not found.

## Data Availability

The original contributions presented in the study are included in the article, further inquiries can be directed to the corresponding authors.
